# Subclinical atherosclerosis, cardiovascular health, and disease risk: is there a case for the Cardiovascular Health Index in the primary prevention population?

**DOI:** 10.1186/s12889-018-5263-6

**Published:** 2018-04-02

**Authors:** Sarah S. Singh, Courtney S. Pilkerton, Carl D. Shrader, Stephanie J. Frisbee

**Affiliations:** 1 Department of Epidemiology & Biostatistics, Schulich School of Medicine & Dentistry, University of Western Ontario, London, ON Canada; 2 Department of Family Medicine, School of Medicine, West Virginia University, Morgantown, WV USA; 3 Departments of Pathology & Laboratory Medicine, and Epidemiology & Biostatistics, Schulich School of Medicine & Dentistry, University of Western Ontario, 1151 Richmond Street, Dental Sciences Building, Room 4041, London, ON N6A 5C1 Canada

**Keywords:** Ankle brachial index, Subclinical atherosclerosis, Cardiovascular Health Index, Framingham Risk Score, Metabolic syndrome, Cardiovascular disease prevention, Cardiovascular risk factors, Primary prevention, NHANES

## Abstract

**Background:**

Current primary prevention guidelines for cardiovascular disease (CVD) prioritize risk identification, risk stratification using clinical and risk scores, and risk reduction with lifestyle interventions and pharmacotherapy. Subclinical atherosclerosis is an early indicator of atherosclerotic burden and its timely recognition can slow or prevent progression to CVD. Thus, individuals with subclinical atherosclerosis are a priority for primary prevention. This study takes a practical approach to answering a challenge commonly faced by primary care practitioners: in patients with no known CVD, how can individuals likely to have subclinical atherosclerosis be easily identified using existing clinical data and/or information provided by the patient?

**Methods:**

Using NHANES (1999–2004), 6091 men and women aged ≥40 years without any CVD comprised the primary prevention population for this study. Subclinical atherosclerosis was determined via ankle-brachial index (ABI) using established cutoffs (subclinical atherosclerosis defined as ABI (0.91–0.99); normal defined as ABI (1.00–1.30)). Three common scores were calculated: the Framingham Risk Score (FRS), the Metabolic Syndrome (MetS), and the Cardiovascular Health Index (CVHI). Logistic regression analysis assessed the association between these scores and subclinical atherosclerosis. The sensitively and specificity of these scores in identifying subclinical atherosclerosis was determined.

**Results:**

In eligible participants, 3.8% had subclinical atherosclerosis. Optimum and average CVHI was associated with decreased odds for subclinical atherosclerosis. High, but not intermediate-risk, FRS was associated with increased odds for subclinical atherosclerosis. MetS was not associated with subclinical atherosclerosis. Of the 3 scores, CVHI was the most sensitive in identifying subclinical atherosclerosis and had the lowest number of missed cases. The FRS was the most specific but least sensitive of the 3 scores, and had almost 10-fold more missed cases vs. the CVHI. The MetS had “middle” sensitivity and specificity, and 10-fold more missed cases vs. the CVHI.

**Conclusions:**

Results from this study suggest that routine administration of the CVHI in a primary prevention population would yield the benefits of identifying patients with existing subclinical CVD not identified through traditional CVD risk factors or scores, and bring physical activity and nutrition to the forefront of provider-patient discussions about lifestyle factors critical to maintaining and prolonging cardiovascular health.

## Background

Cardiovascular disease (CVD), now responsible for 1 in every 4 deaths in the USA and the leading cause of death globally [1], results in enormous societal burden. Primordial and primary prevention remain at the center of strategies [2] to reduce the CVD burden and counter projections that, despite advancements in risk-lowering medication, up to 44% of the USA population will have CVD by 2030 [1, 3], largely due to increased prevalence of obesity and diabetes [2].

Current primary prevention guidelines prioritize risk identification, principally through traditional cardiovascular disease risk factors (obesity, blood pressure, cholesterol, glucose, and smoking), risk stratification using clinical and risk scores, and risk reduction with lifestyle interventions and pharmacotherapy [4–9]. Numerous clinical and risk scores, constructed using algorithms that include varying combinations of traditional CVD risk factors, are available to quantify CVD “risk”; [10] three such scores are the Framingham Risk Score (FRS), the Metabolic Syndrome (MetS), and the Cardiovascular Health Index (CVHI).

The FRS, one of the most well-known and widely used risk scores, was originally developed in 1998 from the Framingham Heart Study cohort to predict 10-year risk of coronary heart disease (CHD) based on age, gender, smoking, cholesterol, diabetes, and blood pressure [10, 11]. It has since been extended to predict absolute risk of a CHD-related event (stroke, myocardial infarction, angina and peripheral vascular disease) in those without clinical CVD [12, 13]. In contrast to the FRS, the MetS is not a predictive score, but the simultaneous presence of multiple traditional CVD risk factors (three of: central obesity; low high-density lipoprotein (HDL) levels; elevated blood pressure; hyperglycemia; hypertriglyceridemia) [14], that may be additive or synergistic in their effect on CVD development [15]. Research has consistently demonstrated a strong association between MetS and increased risk for cardiovascular events and cardiovascular mortality in those with and without known CVD [16, 17]. A comparatively new score, the CVHI was developed in 2010 by the American Heart Association (AHA) as a summative score to quantify cardiovascular health (CVH), as opposed to disease or disease risk [18]. Similar to MetS, the CVHI is an aggregate of well recognized traditional CVD risk factors (blood pressure, total cholesterol, blood glucose, obesity) as well as behavioral factors (smoking, diet, and physical activity). The CVHI was intentionally designed not as a score predictive of CVD or risk of an event, but rather a summative score that quantifies CVH. Multiple studies, in particular Folsom *et al.*, have reported the strong relationship between ideal CVH and favorable outcomes, such as lower incidence of CVD [19]. Importantly for primary prevention, all components of the MetS and CVHI, but not the FRS, are modifiable through lifestyle changes alone [20].

Atherosclerosis is a chronic, inflammatory disease of the arteries that is the most common pathophysiologic process underlying CVD [21]. Like all such processes, atherosclerosis exists along a continuum from subclinical atherosclerosis to patent clinical atherosclerotic vascular disease, can start early in life, and can remain clinically undetected throughout life until an acute event such as myocardial infarction or stroke [21, 22]. Subclinical atherosclerosis is an early indicator of atherosclerotic burden and its timely recognition can slow or prevent the progression to overt CVD [23]. Thus, individuals with subclinical atherosclerosis are a vital priority for primary prevention, but simultaneously a challenge for primary care providers to identify. In this study, we attempt to take a practical approach to answering this challenge commonly faced by primary care practitioners. Specifically: in patients with no known CVD, how can individuals who are likely to have subclinical atherosclerosis, and so in need of prompt primary prevention, be easily identified using existing clinical data and / or information provided by the patient? To accomplish this, we conducted a 2-stage analysis: in the first stage, we determined the association between subclinical atherosclerosis and three common clinical and risk scores. Next, we determined the sensitively and specificity of these scores in identifying those with subclinical atherosclerosis.

## Methods

We conducted a cross-sectional study using data collected as part of the National Health and Nutrition Examination Survey (NHANES). This study was approved as exempt by the University of Western Ontario Research Ethics Board and as non-human subjects research by the West Virginia University Institutional Review Board.

### Participants

NHANES is an ongoing, nationally representative survey conducted by the Centers for Disease Control and Prevention (CDC) that collects data on the health and nutrition status of persons in the USA via interviews and physical examinations. All NHANES data, detailed collection methodology, sampling plans and weights, and analytic guidelines are publicly available [24]. Due to the availability of ankle-brachial index (ABI) (discussed below), data from NHANES surveys (1999–2000, 2001–2002 and 2003–2004) were used for the current study.

All NHANES participants in the above 3 sampling frames were evaluated for eligibility. To achieve a primary prevention population – i.e., those without diagnosed CVD – ineligible participants included those who self-reported a previous diagnosis of CVD (based on an affirmative response to the question, “Has a doctor or other health professional ever told you that you had coronary heart disease, angina (also called angina pectoris), heart attack (also called myocardial infarction), or stroke?”. Participants were also ineligible if their measured ABI was outside the range of 0.90–1.40, as low ABI (≤0.90) indicates peripheral arterial disease (PAD), a systemic manifestation of clinical CVD, and high ABI (≥1.40) indicates incompressible arteries not consistent with atherosclerosis and so not relevant for this study. Thus, eligible participants included individuals with: complete data for ABI; ABI within the range of 0.90–1.40; all variables needed to determine FRS, MetS, and CVHI; and those without a diagnosis of CVD.

For this study of NHANES participants (1999–2004), ABI values were measured on a total of 7571 participants aged ≥40 years. (Note: ABI is not collected in participants < 40 years). A total of 113 subjects were excluded due to high ABI (≥1.40). Of the remaining 7458 participants, a further 1367 were excluded with a diagnosis of CVD or low ABI (≤0.90). Our final sample included a total of 6091 persons, aged ≥40 years, and without a diagnosis of CVD or PAD, which represents a weighted total population of 87,901,942 individuals.

### Outcome measure (dependent variable): subclinical atherosclerosis

The ankle-brachial index (ABI), calculated as the ratio of systolic blood pressure at the ankle to systolic blood pressure at the upper arm, is a non-invasive measurement with well-established cutoff values accepted as indicative of atherosclerosis and/or PAD [25]. While angiography remains the gold standard for detecting atherosclerosis, ABI is an accurate and inexpensive method to detect abnormal limb arterial blood flow and atherosclerotic disease in the peripheral arteries [26, 27]. ABI is significantly associated with the level of subclinical atherosclerosis found in coronary and carotid arteries, making it a valid measure of systemic preclinical disease [28, 29]. Borderline ABI is clinically relevant as it represents subclinical atherosclerosis in systemic vascular beds [30] and is predictive of an increased risk of CVD events [31].

The ABI is the only measure of subclinical atherosclerosis in NHANES. ABI values were automatically calculated as the ratio of the systolic blood pressure of each ankle to the blood pressure in the upper right arm. Mean ABI of the sample was 1.15 (95% CI 1.14–1.16). For the purposes of this study, ABI values were defined according to the 2012 guidelines on measuring and interpreting ABI from the American College of Cardiology and the American Heart Association [32]. Normal ABI was defined as a value of 1.00–1.39. Borderline abnormal ABI, indicative of subclinical atherosclerosis in peripheral arteries, was defined as 0.91–0.99. Mean ABI in the eligible population was 1.15 (95% CI 1.14–1.16) and was non-normally distributed; ABI was only used as a categorical variable in this study.

### Exposure variables (independent variables): FRS, MetS, and CVHI

For this study, three clinical and risk scores were determined for each eligible participant: the Framingham Risk Score (FRS); the Metabolic Syndrome (MetS); and the Cardiovascular Health Index (CVHI).

#### Framingham Risk Score (FRS)

The development and scoring of the FRS has been thoroughly described elsewhere [12]. Components in the FRS include age, total and HDL cholesterol, blood pressure, diabetes and smoking status, all available within NHANES. A total FRS score was calculated for each eligible participant according to the algorithm developed by D’Agostini *et al*. [33] Eligible participants with FRS scores of < 10%, 10–20%, > 20% were classified as low-risk, intermediate-risk and high-risk for a CVD-related event within the next 10 years, respectively.

#### Metabolic Syndrome (MetS)

Classification of MetS was based on the criteria developed by the American Heart Association and the National Heart Lung and Blood Institute [15]. This definition was chosen because it accounted for participants being pharmacologically treated for MetS components as well as clinical parameters, thus capturing all individuals with MetS even if clinical parameters were not elevated at the time of testing. Three of the following five risk factors confirmed the presence of MetS: abdominal obesity (male waist > 40 in. (101.6 cm); female waist > 35 in. (88.9 cm)); elevated triglycerides ≥150 mg/dL (1.69 mmol/L) or on triglyceride lowering medication; low HDL cholesterol (male < 40 mg/dL (1.03 mmol/L); female < 50 mg/dL (1.29 mmol/L)) or on HDL improving medication; blood pressure ≥ 130/≥85 mmHg or on blood pressure lowering medication; fasting glucose ≥100 mg/dL (5.5 mmol/L) or on glucose lowering medication [15]. Eligible participants were classified dichotomously has having MetS (having 3 of the 5 factors above) or not having MetS.

#### Cardiovascular Health Index (CVHI)

The CVHI was developed by the AHA to measure progress towards improving CVH, in contrast to focusing solely on measures of morbidity and mortality [18]. The composite CVHI score is a sum of seven components including blood pressure, cholesterol, blood glucose, physical activity, diet, body mass index, and smoking status [18]. The CVHI was calculated and categorized based on criteria previously published [18] and summarized in Table 1. For this study, the composite CVHI score was calculated on a scale of 0–14, with 2 points awarded for achieving optimum criteria in for that component, 1 point awarded for achieving the average criteria, and 0 points for achieving only inadequate criteria. Thus, an overall CVHI score of 10–14 points indicates optimum CVH, 5–9 points indicates average CVH, and 0–4 points indicates inadequate CVH.

**Table 1:** Definitions for and Questions Used to Determine the Cardiovascular Health Index Score^a^

Component	NHANES Question(s) / Data Used for Component	Inadequate (0 points)	Average (1 point)	Optimum (2 points)
Smoking	Based upon responses to questions: “Have you smoked at least 100 cigarettes in your life?”, “Do you now smoke cigarettes?”, “How long since you last smoked cigarettes?”	Current smoker	Former smoker who quit less than a year ago	Never smoked OR former smoker who quit a year or more ago
Body Mass Index (kg/m^2^)	Calculated based on the height and weight measurements obtained during the clinical examination.	≥30 kg/m^2^	25.0–29.9 ≥ 30 kg/m^2^	< 25.0 ≥ 30 kg/m^2^
Physical Activity	Based upon questions addressing intensity, frequency and duration of physical activity. Moderate intensity activities: those causing “light sweating or a slight to moderate increase in breathing or heart rate.” Vigorous intensity activities: those causing “heavy sweating or large increases in breathing or heart rate”.	None	Moderate intensity (< 150 mins/wk), OR vigorous intensity (< 75 mins/wk)	Moderate intensity (≥150 mins/wk), OR vigorous intensity (≥75 mins/wk) OR Combined intensity (≥150 min/wk)
Diet	Scored as follows: ≥4.5 cups per day of fruits and vegetables (1 point), ≥2 servings of 3.5-oz of fish per week (1 point), ≥3 servings of 1-oz of fiber-rich whole grains per day (1 point), < 1500 mg sodium per day (1 point), and < 450 kcal of added sugar in sugar-sweetened beverages per week (1 point).	0–1 diet points	2–3 diet points	4–5 diet points
Total Cholesterol (mg/dL)^b^	Determined according to procedures described in the NHANES Laboratory/Medical Technologists Procedures Manual for the collecting and storing blood samples, and for laboratory processing of plasma lipids and glucose [49].	≥240 mg/dL [≥6.21 mmol/L]	200–239 mg/dL OR achieved goal on cholesterol lowering medication [5.17–6.18 mmol/L)	< 200 mg/dL and not on cholesterol lowering medication [< 5.17 mmol/L]
Blood pressure (mmHg)	Measured by qualified technicians after the subjects had been sitting quietly for 5 min. Blood pressure measurement were taken at least 3 times on each subject and the average of these values were used for this study.	SBP ≥140 mmHg OR DBP ≥90 mmHg	SBP 120–139 mmHg OR DBP 80–89 mmHg OR achieved goal on blood pressure lowering medication	SBP < 120 mmHg and DBP < 80 AND not on blood pressure lowering medication
Fasting blood glucose (mg/dL)^b^	Determined according to procedures described in the NHANES Laboratory/Medical Technologists Procedures Manual for the collecting and storing blood samples, and for laboratory processing of plasma lipids and glucose [49].	≥126 mg/dL [≥7.0 mmol/L]	100–125 mg/dL OR achieved goal on glucose lowering medication [5.5–6.99 mmol/L]	< 100 mg/dL AND not on glucose lowering medication [< 5.5 mmol/L]

^a^Components, cut-off values, and scoring criteria as defined in Lloyd-Jones et al. (2010) [18]

^b^SI units shown in square brackets

### Covariates

For study participants, demographic variables abstracted from NHANES included: age; race/ethnicity; sex; education; and smoking. Age was categorized based on the 2000 USA projected population age distribution categories: 40–59 years; 60–74 years; and 75 years and over. Race was categorized as: non-Hispanic white; non-Hispanic black; or other. Education was categorized as high school education vs. no high school education. Smoking was based on a positive response to the question: “Have you smoked at least 100 cigarettes in your life?”

### Statistical analysis

NHANES guidelines were used to merge data from years 1999–2004 and to correctly apply sampling weights, primary sampling units, and strata. For univariate analyses, descriptive statistics were conducted to produce weighted estimates of proportions (%) and 95% confidence intervals (CI). For multivariable analyses, all analyses were conducted using survey procedures to account for NHANES survey weights and sampling design.

To assess the relationship between the FRS, MetS, CVHI and subclinical atherosclerosis, separate, independent logistic regression models were estimated. All models were structured to produce estimates adjusted for all covariates listed above. Interactions were tested independently between FRS, MetS, CVHI and age, sex, race. Tested interactions were not significant for FRS, MetS or CVHI with age, sex or race independently (all *p* > 0.05, data not shown) and so stratified analyses were not considered further.

To assess the ability of FRS, MetS, and CVHI to correctly identify individuals with subclinical atherosclerosis, sensitivity and specificity analyses were performed. Sensitivity (probability of correctly detecting true-positive results) and specificity (probability of correctly detecting true-negative results) based upon the on the selected clinical scores (FRS, MetS, and CVHI) were calculated using standard formulae. The values of sensitivity and specificity are reported for weighted estimates of the study population. Finally, traditional CVD risk factors (blood pressure, total cholesterol, fasting blood glucose, and body mass index) were examined for individuals with false-negative results – i.e., those who had subclinical atherosclerosis but were labelled as healthy or low-risk by the FRS, MetS, or CVHI. For each score, the weighted mean for each traditional CVD risk factor was determined for the group of individuals with false-negative results (missed cases of subclinical atherosclerosis).

All statistical analyses were performed using Stata Statistical Software Release 14 (College Station, TX: StataCorp LP). As all analyses were conducted using survey procedures to account for NHANES survey weights and sampling design, all results are reported as weighted.

## Results

Characteristics in the eligible population are reported in Table 2. Most eligible participants were aged 40–49 (40.6%), non-Hispanic white (76.9%), smokers (52.3%), and had a high school education (81.7%). Exactly 3.4% of the population had subclinical atherosclerosis, as defined by borderline ABI (0.91–0.99), with the remaining 96.6% of the study population classified as normal ABI (1.00–1.39) and so free of subclinical atherosclerosis.

**Table 2:** Demographic characteristics of the eligible population from the National Health and Nutrition Examination Survey (1999–2004)

	Weighted Proportion^a^
Age category (years)	
40–49	40.6 (38.5–42.8)
50–64	38.7 (36.8–39.9)
65 and older	20.7 (19.5–21.9)
Sex	
Male	47.6 (46.1–49.0
Female	52.4 (50.9–53.9)
Race/Ethnicity	
Non-Hispanic White	76.9 (73.4–80.0)
Non-Hispanic Black	9.3 (7.5–11.4)
Other	13.9 (10.9–17.3)
Education	
Less than High School Education/no GED (General Education Diploma)	18.3 (16.6–20.0)
High School Education/GED or more	81.7 (79.9–83.4)
Smoking	
No	47.6 (45.7–49.5)
Yes (smoked at least 100 cigarettes in life)	52.3 (50.4–54.2)

^a^Weighted proportion of the eligible population (*n* = 6091) after applying sampling weights to the study sample (% (95% CI))

Table 3 summarizes the distribution of the outcome and exposure variables between participants with and without subclinical atherosclerosis. Of note, the proportion of those classified as high-risk using the FRS was approximately doubled in the subclinical atherosclerosis group (19.4%) as compared to those without subclinical atherosclerosis (8.5%). There was also a higher proportion of MetS (37.2% vs 24.6%) and inadequate CVHI (15.8% vs 11.6%) health in those with subclinical atherosclerosis compared to those without subclinical atherosclerosis, respectively.

**Table 3:** Characteristics of the outcome and exposure variables in the eligible population^a^

	All	Subclinical Atherosclerosis^b^	No Subclinical Atherosclerosis^c^	Statistical Significance^d^
Ankle Brachial Index (ABI)	–	3.4 (2.9–4.0)	96.6 (95.9–97.1)	
Framingham Risk Score				
Low risk (< 10%)	59.1 (57.8–60.4)	53.1 (0.45–61.4)	59.3 (58.0–60.6)	§
Intermediate risk (10%–20%)	32.1 (30.6–33.5)	27.5 (20.1–36.4)	32.2 (30.7–33.7)	
High risk (> 20%)	8.9 (8.0–9.8)	19.4 (14.0–26.4)	8.5 (7.7–9.3)	
Metabolic Syndrome				
No	75.0 (73.9–76.6)	62.8 (54.5–70.4)	75.4 (73.7–77.0)	§
Yes (3 or more components)	25.0 (23.4–26.7)	37.2 (29.6–45.5)	24.6 (23.0–26.3)	
Cardiovascular Health Index^e^				
Optimum overall health (12–14 points)	12.0 (10.3–13.9)	4.4 (1.5–12.5)	13.2 (11.5–15.2)	
Average overall health (8–11 points)	75.7 (73.9–77.4)	79.8 (72.7–85.4)	75.2 (73.3–77.0)	
Inadequate overall health (0–7 points)	12.3 (11.0–13.7)	15.8 (11.5–21.4)	11.6 (10.3–13.0)	§

^a^Proportion estimates of the eligible population after applying sampling weights to the study sample (% (95% CI))

^b^Defined as borderline ABI (0.91–0.99)

^c^Defined as normal ABI (1.00–1.39)

^d^Pearson’s chi squared test for comparing differences in proportions between normal ABI and borderline ABI

^e^Categorization criteria and scoring for the Cardiovascular Health Index is described in Table 1

^§^Indicates statistical significance at the *p* < 0.05 level

**Fig. 1:**
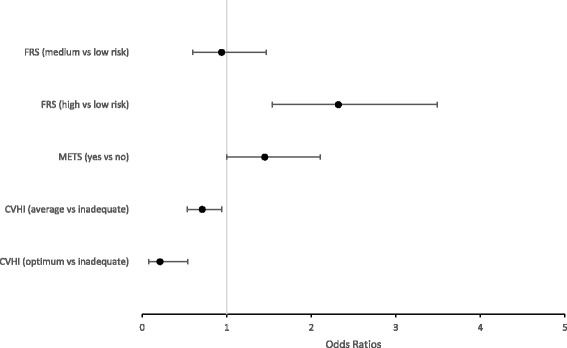
Results from logistic regression analysis assessing the association between clinical and risk scores and subclinical atherosclerosis^‡^. ^‡^Results displayed as the Adjusted Odds Ratio and 95% Confidence Interval, with analysis conducted while applying sampling weights to the study sample; Framingham Risk Score models adjusted for race and education; Metabolic syndrome models adjusted for sex, age, race, smoking, and education; Cardiovascular Health Index models adjusted for sex, age, race, and education

In Fig. 1, results from the logistic regression assessing the association between FRS, MetS and CVHI and subclinical atherosclerosis in the eligible population are displayed. Compared to individuals classified as low-risk by the FRS, individuals classified as intermediate-risk were not more likely to have subclinical atherosclerosis, though individuals classified as high-risk by the FRS were more than twice as likely to have subclinical atherosclerosis (OR 2.31; 95% CI 1.53–3.49). The presence of MetS was only marginally associated with increased likelihood of subclinical atherosclerosis (for individuals with MetS compared to those without MetS OR 1.45; 95% CI 1.00–2.11). Finally, in examining the results for CVHI, it is noted that, compared to individuals with inadequate overall CVHI, individuals with average overall CVHI were 29% less likely to have subclinical atherosclerosis (OR 0.71; 95% CI 0.53–0.94), and individuals with optimum CVHI were almost 80% less likely to have subclinical atherosclerosis (OR 0.21; 95% CI 0.08–0.54). Additionally, across all models, the odds of having subclinical atherosclerosis were highest for those participants aged 65 years and older, females, non-Hispanic black race, and without a high school education (results not shown).

Results from the sensitivity and specificity analysis are reported in Table 4. Of the scores evaluated, high-risk FRS (vs. low-risk) was both least sensitive (26.6%) and most specific (87.4%) in identifying individuals with subclinical atherosclerosis. Intermediate-risk FRS (vs. low-FRS) had slightly better sensitivity (33.9%), but also had lower specificity (64.9%). In contrast, average CVHI (vs. optimum CVHI) was most sensitive (94.8%) but least specific (14.9%). Inadequate CVHI (vs. optimum CVHI) had lower sensitivity (78.0%) but higher specificity (53.2%). MetS had better sensitivity than both intermediate- and high-risk FRS (36.7%), but not higher than either average or inadequate CVHI, but had higher specificity than all but high-risk FRS at 75.3%. Table 5 details the weighted number and proportion of participants who had subclinical atherosclerosis but were not identified (missed) by the FRS, MetS or CVHI score. The CVHI score achieved the fewest missed cases of subclinical atherosclerosis; the FRS and MetS had a similar number of missed cases, almost 10-fold higher than the CVHI. The weighted mean of traditional cardiovascular disease risk factors for these cases, for each score, is also reported in Table 5. These CVD risk profiles are similar for each of the three scores and, on average, fall within the normal range for the risk factor – i.e., would not be identified as needing treatment. That is, for many of these missed cases, subclinical atherosclerosis is present in the absence of being identified as at-risk by either the FRS, MetS, or CVHI, and without abnormal traditional CVD risk factors.

**Table 4:** Sensitivity and specificity of clinical and risk scores to identify individuals with subclinical atherosclerosis^a^

	Framingham Risk Score^d^		Metabolic Syndrome^e^	Cardiovascular Health Index^f^	
	Intermediate Risk	High Risk	Yes	Average	Inadequate
Sensitivity^b^	33.9% (33.8─33.9)	26.6% (26.5─26.6)	36.7% (36.6─36.7)	94.8% (94.7─94.8)	78.0% (77.8─78.1)
Specificity^c^	64.9% (64.8─64.9)	87.4% (87.3─87.4)	75.3% (75.2─75.3)	14.9% (14.8─14.9)	53.2% (53.1─53.3)

^a^Proportion estimates of the eligible population after applying sampling weights to the study sample (% (95% CI))

^b^Sensitivity: probability of correctly detecting true positive results (individuals who *do* have subclinical atherosclerosis)

^c^Specificity: probability of correctly detecting true negative results (individuals who *do not* have subclinical atherosclerosis)

^d^Framingham Risk Score: Intermediate (10–20% 10-year risk) or high risk (> 20% 10-year risk) vs. low risk (referent value; < 10% 10-year risk)

^e^Metabolic syndrome: Presence of metabolic syndrome (3 or more risk factors) vs. no metabolic syndrome (referent value; < 3 risk factors)

^f^Cardiovascular Health Index (CVHI): Average CV health (5–9 total points) or inadequate CV health (0–4 total points) vs. optimum CV health (reference value; 10–14 total points)

**Table 5:** Number, proportion, and mean values for traditional cardiovascular disease risk factors^a^ of individuals misclassified as false-negative (cases of missed subclinical atherosclerosis)

Framingham Risk Score	
Missed Subclinical Atherosclerosis (Low Risk FRS)^b^	Risk Factor Profile of Missed Individuals with Subclinical Atherosclerosis
n: 1,600,000 1.8% (1.5─2.1)	n: 1,600,000 SBP: 137.4 mmHg (130.9–143.9) DBP: 69.8 mmHg (66.7–72.9) Total cholesterol: 203.8 mg/dL (195.8–211.9) [5.27 mmol/L (5.06–5.48)] Fasting glucose: 105.6 mg/dL (94.5–116.8) [5.9 mmol/L (5.3–6.5)] BMI: 28.6 kg/m^2^ (27.1–30.1)
Metabolic Syndrome	
Missed Subclinical Atherosclerosis (No MetS)^c^	Risk Factor Profile of Missed Individuals with Subclinical Atherosclerosis
n: 1,900,000 2.1% (1.7─2.6)	n: 1,900,000 SBP: 139.7 mmHg (134.4–144.9) DBP: 71.7 mmHg (69.6–73.8) Total cholesterol: 212.8 mg/dL (204.9–220.6) [5.51 mmol/L (5.31–5.71)] Fasting glucose: 93.8 mg/dL (90.8–96.8) [5.2 mmol/L (5.0–5.4)] BMI: 27.7 kg/m^2^ (26.6–28.7)
Cardiovascular Health Index	
Missed Subclinical Atherosclerosis (Optimum CVHI)^d^	Risk Factor Profile of Missed Individuals with Subclinical Atherosclerosis
n: 110,000 0.1% (0.01─0.4)	n: 110,000 SBP: 120.9 mmHg (101.8–140.1) DBP: 68.5 mmHg (69.9–71.7) Total cholesterol: 175.5 mg/dL (158.1–195.1) [4.54 mmol/L (4.09–5.03)] Fasting glucose: 99.8 mg/dL (93.8–105.8) [5.5 mmol/L (5.2–5.9)] BMI: 28.6 kg/m^2^ (21.3–23.5)

^a^Number and proportion estimates of the eligible population after applying sampling weights to the study sample (% (95% CI)); SI units shown in square brackets

^b^Classified as “low risk” by the Framingham Risk Score (< 10% 10-year risk) but with subclinical atherosclerosis present

^c^Classified as not having the metabolic syndrome (< 3 risk factors) but with subclinical atherosclerosis present

^d^Classified as having “optimum” health by the Cardiovascular Health Index (CVHI; 10–14 total points) but with subclinical atherosclerosis present

## Discussion

In this study of a primary prevention population derived from a nationally representative sample, we sought to understand (A) the association between subclinical atherosclerosis and the 3 clinical scores FRS, MetS, and CVHI; and (B) the ability of these different scores to identify individuals with subclinical atherosclerosis. The two key observations reported in this study are:

1. Optimum and average CVHI was associated with decreased odds for subclinical atherosclerosis. High, but not intermediate-risk, FRS was associated with increased odds for subclinical atherosclerosis. MetS was not associated with subclinical atherosclerosis.
2. Of the 3 clinical scores, CVHI was the most sensitive in identifying cases of subclinical atherosclerosis and had the lowest number of missed cases. The FRS was the most specific but least sensitive of the 3 scores, and had almost 10-fold more missed cases compared to the CVHI. The MetS had “middle” sensitivity and specificity, and 10-fold more missed cases compared to the CVHI.

To our knowledge, this study is the first to report these observations in a nationally representative sample, and in a primary prevention population.

### Comparison to other studies

Our findings are consistent with both current knowledge of atherogenesis as well as findings from multiple, previous studies. Prior studies of low ABI, diagnostic of PAD, have reported strong associations between low ABI and MetS [34] and the FRS in individuals with or without CVD or diabetes [35]. In a recent study of participants in the Multi-Ethnic Study of Atherosclerosis (MESA), it was reported that adults with optimum or intermediate CVHI were 71% and 43%, respectively, less likely to have subclinical atherosclerosis [36]. Additionally, investigators from the Jackson Heart Study cohort have recently described the relationship between CVHI and low ABI, establishing a 34% increased odds of PAD in African-Americans with inadequate CVHI (OR 1.34; 95% CI 1.11–1.63) [37]. Our findings extend these observations by linking CVH, as measured by the CVHI, to subclinical atherosclerosis. The importance of the association with subclinical atherosclerosis is that it identifies and highlights individuals most at need for primary prevention measures so as to delay atherogenesis and prevent the development of clinical CVD. As has been previously discussed by others, borderline ABI represents a critical point in the inception of atherosclerotic disease when treatment and risk factor modification may significantly reduce the progression of atherosclerosis and consequent occurrence of CVD [30, 38].

Our results are also consistent with a growing body of literature describing the occurrence of CVD and cardiovascular events in those without traditional CVD risk factor profiles; for example, a recent study by Fernandez-Friera *et al.*, reported that subclinical atherosclerosis existed in almost 50% of participants free of traditional CVD risk factors [39]. Our observations extend these previous reports to a primary prevention population: specifically, that, for a portion of the CVD-free participants in this study, subclinical atherosclerosis was present in the absence of being identified as at-risk by either the FRS, MetS, or CVHI, and without abnormal traditional CVD risk factors.

### Implications for primary prevention: the case for the CVHI as a routine part of primary care

More than $$ \raisebox{1ex}{$1$}\!\left/ \!\raisebox{-1ex}{$3$}\right. $$ of USA adults have at least one form of CVD [40] and almost $$ \raisebox{1ex}{$2$}\!\left/ \!\raisebox{-1ex}{$3$}\right. $$ of USA adults have at least one traditional CVD risk factor [41]. Thus, it is a constant challenge for primary care providers to readily identify patients most in need of prompt primary prevention: those patients without existing CVD and / or who may not yet meet pharmacologic treatment thresholds for traditional CVD risk factors, but are likely to have subclinical atherosclerosis and, thus, prompt primary prevention would prolong cardiovascular health and postpone – or prevent – the development of clinical CVD. Current USA guidelines for the assessment and treatment of traditional CVD risk factors include the calculation of the FRS (U.S. Preventative Services Task Force) [9] or the Pooled Cohort Equation, derived from the FRS (American Heart Association) [5]. (Note: the Pooled Cohort Equation was not included in this study as (a) it is only applicable in non-Hispanic whites and African-Americans [5], which would have resulted in the exclusion of 30% of the study population sample from “other” races; and (b) there remain ongoing debates about the accuracy of the calibration of this score [42–46].) However, results from this study suggest a provocative question: in the primary prevention population, is the CVHI a more effective tool to identify individuals in need of prompt primary prevention?

The case for the CVHI is threefold. First, with the inclusion of the lifestyle components physical activity and nutrition – the only score to do so – use of the CVHI would bring these factors systematically to the fore, which is inherently more consistent with current guidelines that specify lifestyle management as the first stage in CVD primary prevention [4]. Second, in light of recent evidence that traditional CVD risk factors fail to identify substantial proportions of individuals with subclinical CVD [39], it is intriguing to contemplate that the apparent effectiveness of the CVHI in identifying subclinical CVD may be related to the inclusion of the physical activity and nutrition components, which may serve as a proxy for factors associated with vascular health not captured by traditional CVD risk factors. Third and finally, as the CVHI is a report-card score, measuring individuals against a standard of ideal health, and not a predictive score such as the FRS which is designed to predict the likelihood of a particular event within a defined time period, the CVHI largely avoids the challenges of its accuracy and calibration changing in different population subsets. That is, the simplicity of the CVHI may allow it to be universally applicable across large and diverse populations.

Thus, as risk-based scores are increasingly being incorporated into electronic health records [44, 47], results from this study suggest that systematic inclusion of the CVHI warrants earnest consideration. Given the overlap in elements between risk-based CVD scores, such as the FRS and the Pooled Cohort Equations, extension to the CVHI would not be impracticable. While further study is needed, results from this study suggest that routine inclusion would yield the benefits of identifying patients with existing subclinical CVD, and bringing physical activity and nutrition to the forefront of provider-patient discussions about lifestyle factors critical to maintaining and prolonging cardiovascular health.

### Strengths and limitations

The primary strength of this study is the use of NHANES, a nationally representative sample. A key limitation is the restricted time period for ABI measurements (1999–2004) and the cross-sectional nature of NHANES. Additionally, as NHANES did not include any questions on symptomaticity in their subject questionnaires, temporal inferences and the prevalence of undiagnosed CVD or PAD could not be evaluated. It is also noted that NHANES only included ABI measures on individuals ≥40 years of age. Future studies should assess the comparative utility of the CVHI in younger age groups and pre-menopausal women, where risk prediction is challenging but, simultaneously, where primary prevention would likely yield larger gains.

Three additional caveats are warranted. First, as described in the Methods section above, ABI in this study was used to identify NHANES participants with subclinical atherosclerosis; ABI is the only measurement of subclinical atherosclerosis included in NHANES. While ABI is strongly associated with other measures of subclinical atherosclerosis such as carotid intima-media thickness or the coronary artery calcification score [28], these scores assess atherosclerotic burden in different vascular beds so it is possible that any one of these measures does not capture all cases of subclinical atherosclerosis. Future studies could assess whether the observations reported here are consistent across different – or a combination of – measures of atherosclerotic burden. Second, in this study ABI was not used as a screening tool, which is not recommended under current USA-based guidelines [48], nor did this study attempt to evaluate the ABI as a screening tool. ABI is also not the singular, “gold-standard” clinical test for atherosclerosis and was not used as such in this study. Third, the FRS, but not the Pooled Cohort Equations, was included in this study for the practical reasons described above; the predictive accuracy of either score was not assessed in this study.

## Conclusions

Results from this study suggest that routine administration of the CVHI in a primary prevention population would yield the benefits of identifying patients with existing subclinical CVD not identified through traditional CVD risk factors or scores, and bring physical activity and nutrition to the forefront of provider-patient discussions about lifestyle factors critical to maintaining and prolonging cardiovascular health. We anticipate that the findings from our study will encourage practitioners to monitor cardiovascular health even in patients classified as low-risk by traditional disease risk measures. It is well known that a large proportion of CVD events occur in individuals classified as low-risk; routine CVHI assessment may be positive step toward the goal of reducing CVD events in the primary prevention population.

## Abbreviations

ABI: Ankle-brachial index
AHA: American Heart Association
BMI: Body mass index
CAC: Coronary artery calcification
CDC: Centers for Disease Control and Prevention
CHD: Coronary heart disease
CI: Confidence interval
CV: Cardiovascular
CVD: Cardiovascular disease
CVH: Cardiovascular health
CVHI: Cardiovascular Health Index
FRS: Framingham Risk Score
MESA: Multi-Ethnic Study of Atherosclerosis
MetS: Metabolic syndrome
NHANES: National Health and Nutrition Examination Survey
OR: Odds ratio
PAD: Peripheral artery disease
USA: United States of America
